# [Expression of Concern] Reciprocal negative feedback loop between EZH2 and miR-101-1 contributes to miR-101 deregulation in hepatocellular carcinoma

**DOI:** 10.3892/or.2025.8997

**Published:** 2025-09-25

**Authors:** Da Huang, Xiaobei Wang, Chunbo Zhuang, Wuhe Shi, Mu Liu, Qiming Tu, Detai Zhang, Lihua Hu

Oncol Rep 35: 1083–1090, 2016; DOI: 10.3892/or.2015.4467

Following the publication of this paper, it was drawn to the Editor's attention by a concerned reader that, for the Transwell assay data shown in Fig. 5B on p. 1088, the rightmost panels in the top and the bottom rows appeared to contain a small overlapping section, suggesting that data which were intended to show the results of differently performed experiments had been derived from the same original source.

The authors were contacted by the Editorial Office to offer an explanation for this apparent anomaly in the presentation of the data in this paper; however, up to this time, no response from them has been forthcoming. Owing to the fact that the Editorial Office has been made aware of potential issues surrounding the scientific integrity of this paper, we are issuing an Expression of Concern to notify readers of this potential problem while the Editorial Office continues to investigate this matter further.

